# DJ-1 Can Replace FGF-2 for Long-Term Culture of Human Pluripotent Stem Cells in Defined Media and Feeder-Free Condition

**DOI:** 10.3390/ijms22115954

**Published:** 2021-05-31

**Authors:** Julee Kim, Sangki Baek, Yean Ju Hong, Michelle Novais de Paula, Musharrat Jahan Prima, Yeon-Mok Oh, Sun-Shin Cha, Jeong Tae Do, Yeon Jin Jang, Han Choe

**Affiliations:** 1 Department of Physiology, Bio-Medical Institute of Technology, University of Ulsan College of Medicine, Asan Medical Center, Seoul 05505, Korea; juleekim119@gmail.com (J.K.); sangkib64@gmail.com (S.B.); Michellenovaisko@gmail.com (M.N.d.P.); prima.musharrat@gmail.com (M.J.P.); yjjang@amc.seoul.kr (Y.J.J.); 2 Department of Stem Cell and Regenerative Biotechnology, KU Institute of Science and Technology, Konkuk University, Seoul 05029, Korea; ndhong7@gmail.com (Y.J.H.); dojt@konkuk.ac.kr (J.T.D.); 3 Department of Pulmonary and Critical Care Medicine, Asan-Minnesota Institute for Innovating Transplantation, University of Ulsan College of Medicine, Asan Medical Center, Seoul 05505, Korea; ymoh55@amc.seoul.kr; 4 Department of Chemistry & Nanoscience, Ewha Womans University, Seoul 03760, Korea; chajung@ewha.ac.kr

**Keywords:** hPSC, DJ-1, FGF-2, defined media, feeder-free

## Abstract

Conventional human pluripotent stem cell (hPSC) cultures require high concentrations of expensive human fibroblast growth factor 2 (hFGF-2) for hPSC self-renewal and pluripotency in defined media for long-term culture. The thermal instability of the hFGF-2 mandates media change every day, which makes hPSC culture costly and cumbersome. Human DJ-1 (hDJ-1) can bind to and stimulate FGF receptor-1. In this study, for the first time, we have replaced hFGF-2 with hDJ-1 in the essential eight media and maintained the human embryonic stem cells (hESCs), H9, in the defined media at feeder-free condition. After more than ten passages, H9 in both groups still successfully maintained the typical hESC morphology and high protein levels of pluripotency markers, SSEA4, Tra1-60, Oct4, Nanog, and ALP. DNA microarray revealed that more than 97% of the 21,448 tested genes, including the pluripotency markers, Sox2, Nanog, Klf4, Lin28A, Lin28B, and Myc, have similar mRNA levels between the two groups. Karyotyping revealed no chromosome abnormalities in both groups. They also differentiated sufficiently into three germ layers by forming in vitro EBs and in vivo teratomas. There were some variations in the RT-qPCR assay of several pluripotency markers. The proliferation rates and the mitochondria of both groups were also different. Taken together, we conclude that hDJ-1 can replace hFGF-2 in maintaining the self-renewal and the pluripotency of hESCs in feeder-free conditions.

## 1. Introduction

Human pluripotent stem cells (hPSCs) such as human embryonic stem cells (hESC) or human-induced pluripotent stem cells (hiPSC) have high potential as therapeutic agents in regenerative medicine. One of the crucial challenges for these applications is maintaining and expanding hPSCs in an undifferentiated state without acquiring genetic abnormalities in the feeder-free and xeno-free conditions for the long term. Conventional hPSC cultures require high concentrations of expensive human fibroblast growth factor 2 (hFGF-2) for self-renewal and pluripotency of hPSC in defined media and feeder-free condition for long-term culture [1,2,3,4,5]. However, hPSC culture media need to be changed every day partly due to the hFGF-2 thermal instability in solution at 37 °C [6,7], which makes hPSC culture costly and cumbersome.

Human DJ-1 (hDJ-1), also known as PARK-7, is a 189-residue homodimer that was first cloned as an oncogene induced by growth stimuli and transforming mouse NIH3T3 cells [8]. hDJ-1 attracted great attention due to its direct implication in autosomal recessive early-onset Parkinson’s disease [9]. hDJ-1 is ubiquitously expressed in various human tissues and has multiple enzymatic functions such as a protease [10], a glyoxalase [11], a deglycase [12], esterase [13], and even a molecular chaperone [14,15]. Under normal conditions, it is located predominantly in the cytoplasm and, to a lesser extent, in the nucleus and mitochondria. It translocates to the mitochondrion and subsequently to the nucleus and increases the cytoprotective effect under oxidative stress [16]. hDJ-1 is also secreted into the extracellular space where the protein binds to fibroblast growth factor receptor 1 (FGFR-1), even though it does not have sequence homology with the FGF family [17]. hDJ-1′s binding site to FGFR-1 is different from that of hFGF-2 [17]. 

To date, there has been no report of hDJ-1′s effects on hPSC self-renewal and pluripotency. Therefore, we were interested in whether hDJ-1 without hFGF-2 can maintain the hPSCs’ self-renewal and pluripotency in feeder-free condition or not. This study used the defined media Essential 8 (E8) containing either hFGF-2 or hDJ-1 and maintained H9 hESC under feeder-free conditions for more than ten passages. The cells maintained by hFGF-2 and hDJ-1 were compared for their morphology, pluripotency, proliferation rate, ultrastructure, and chromosome integrity.

## 2. Result

**Figure 1:**
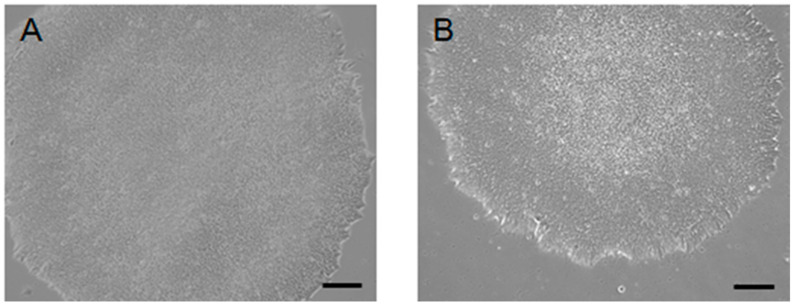
Morphological analysis of H9 hESCs after ten passages cultured in homemade E8 media under a feeder-free condition. The media contained either hFGF-2 (**A**) or hDJ-1 (**B**). Scale bars indicate 100 µm.

**Figure 2:**
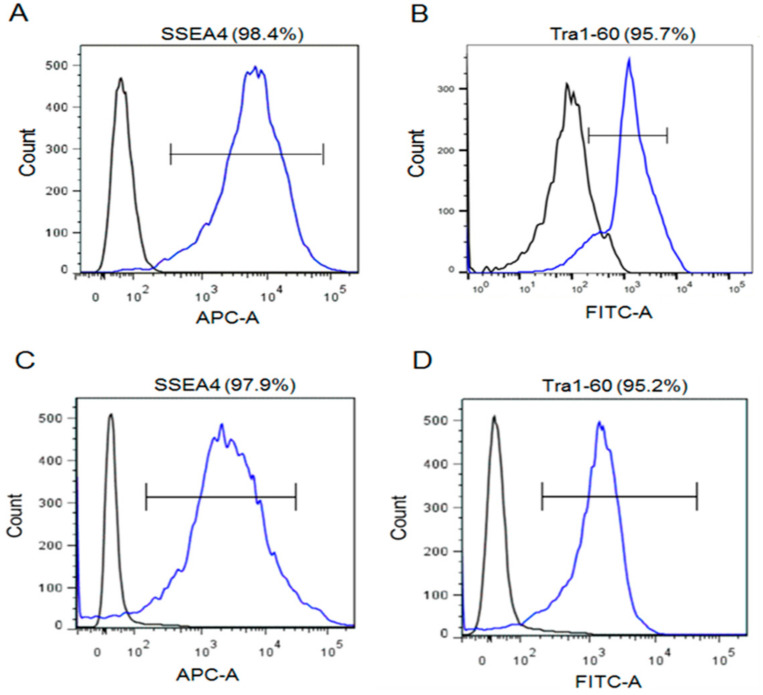
FACS analysis of H9 hESCs after 10 passages cultured in homemade E8 medium containing either hFGF-2 (**A**,**B**) or hDJ-1 (**C**,**D**) for the pluripotency marker SSEA4 (**A**,**C**) or Tra1-60 (**B**,**D**).

**Figure 3:**
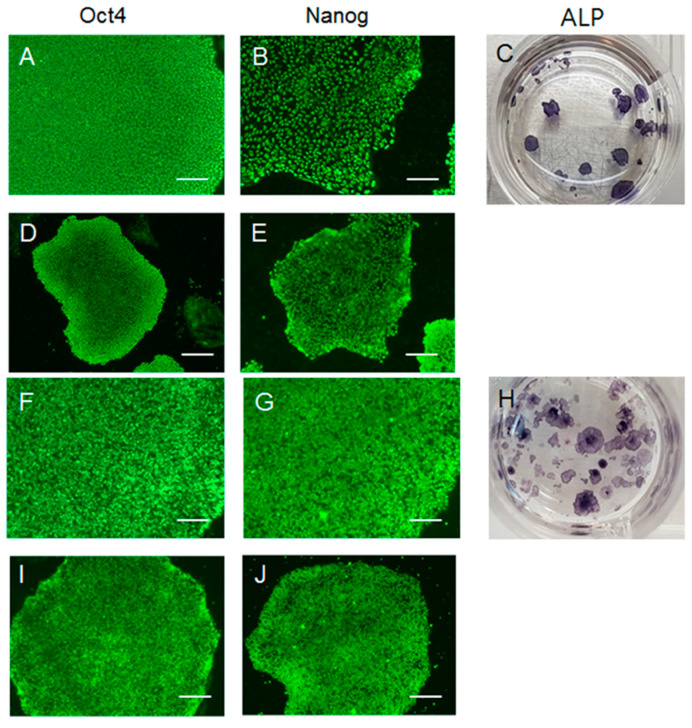
Immunofluorescent staining of H9 hESCs after ten passages cultured in homemade E8 medium containing either hFGF-2 (**A**–**E**) or hDJ-1 (**F**–**J**) stained with pluripotency marker Oct4 (**A**,**D**,**F**,**I**) and Nanog (**B,E**,**G**,**J**). ALP was stained in H9 hESCs after ten passages cultured in homemade E8 medium containing hFGF-2 (**C**) or hDJ-1 (H). Scale bars indicate 100 µm (**A**,**B**,**F**,**G**) and 200 µm (**D**,**E**,**I**,**J**).

H9 hESCs were cultured in homemade E8 media containing either hFGF-2 or hDJ-1. For more than ten passages, H9 hESCs in both conditions maintained similar flat morphologies and well-defined boundaries, which are typical hESCs characteristics (Figure 1). Without either hFGF-2 or hDJ-1, the hESCs did not survive more than one passage (data not shown). FACS analysis was performed using antibodies targeting two well-known pluripotency markers, SSEA4 and Tra1-60, on the cell surface of H9 hESCs passaged ten times in homemade E8 medium (Figure 2). Both hFGF-2 and hDJ-1 maintained the high protein level of the two pluripotency markers. SSEA4 and Tra1-60 were expressed in 98% and 95%, respectively, of the cells grown by either hFGF-2 or hDJ-1. The protein levels of three other well-known pluripotency markers, Oct4, Nanog, and alkaline phosphatase (ALP), were also examined (Figure 3). Immunostaining of Oct4 and Nanog for hESCs after ten passages demonstrated that both makers’ high expression was well-maintained by hFGF-2 or hDJ-1. ALP staining also showed good expression of ALP in both H9 cells. 

**Figure 4:**
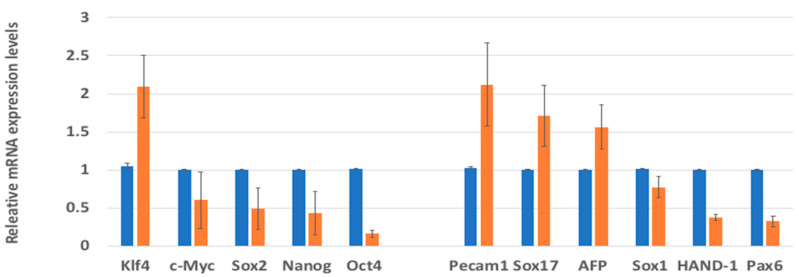
RT-qPCR analysis of H9 hESC cultured in homemade medium containing 100 ng/mL of hFGF-2 (blue bar) or hDJ-1 (orange bar) after ten passages.

The messenger ribonucleic acid (mRNA) expression levels of several pluripotent and differentiation genes were compared in the H9 hESCs maintained with hFGF-2 or hDJ-1 using the real-time quantitative polymerase chain reaction (RT-qPCR) technique (Figure 4). Overall, the mRNA expression levels by hDJ-1 were similar to or sometimes lower than those by hFGF-2. The mRNA expression levels of the pluripotency marker, Klf4, and the differentiation markers, Sox17 and AFP, were similar in both cell groups. However, the mRNA expression level of the pluripotency markers, Oct4, Sox2, c-Myc, and Nanog, and the differentiation markers, Pax6, Sox1, CD31, and HAND-1, were lower in H9 hESCs grown with hDJ-1 than by hFGF-2. We also found the mRNA expression level of hDJ-1 was lower in H9 hESCs grown with hDJ-1 than those with hFGF-2.

**Figure 5:**
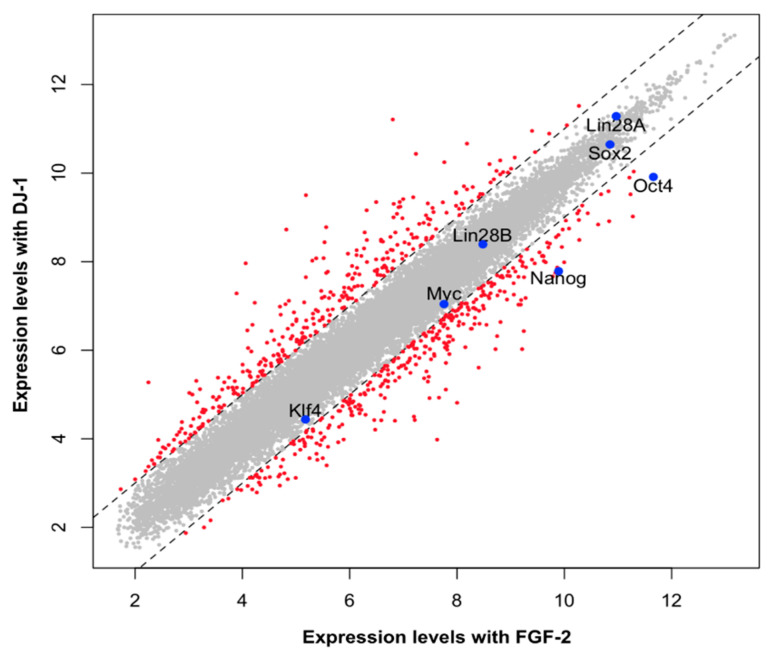
Microarray analysis of H9 hESC cultured in homemade medium containing hFGF-2 or hDJ-1 for ten passages.

The cells were subjected to microarray analysis of 21,448 probes to compare the gene expressions of H9 hESC by hFGF-2 and hDJ-1 more comprehensively (Figure 5 and Table S3). The mRNA expression of more than 97% of the tested genes showed differences of less than 2-fold between the two groups. The pluripotency markers including Sox2, Nanog, Klf4, Lin28A, Lin28B, and Myc expressed similar mRNA levels in H9 hESCs cultured with hFGF-2 and hDJ-1. However, some genes, especially two pluripotency markers, Oct4 and Nanog, showed lower mRNA expression levels in H9 hESCs cultured with hDJ-1. Gene Ontology (GO) and Kyoto Encyclopedia of Genes and Genomes (KEGG) pathway gene-enrichment and functional annotation analysis were performed for 639 probes that showed more than 2-fold differences (Supplementary Figures S1 and S2). Notably, the PI3K–Akt signaling pathway has been most significantly different. 

**Figure 6:**
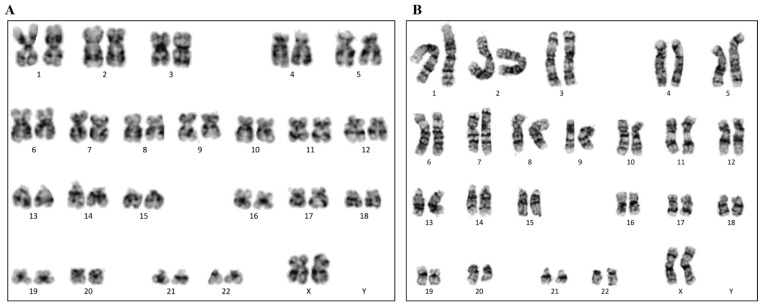
Karyotyping of H9 hESCs p55 after ten passages cultured in homemade E8 medium containing hFGF-2 (**A**) or hDJ-1 (**B**).

After ten passages of cultures in homemade E8 media containing either hFGF-2 or hDJ-1, any chromosome abnormalities of H9 hESCs were assessed by karyotyping (Figure 6). A minimum of 20 metaphase spreads was analyzed. All of the cells showed normal chromosomes. 

**Figure 7:**
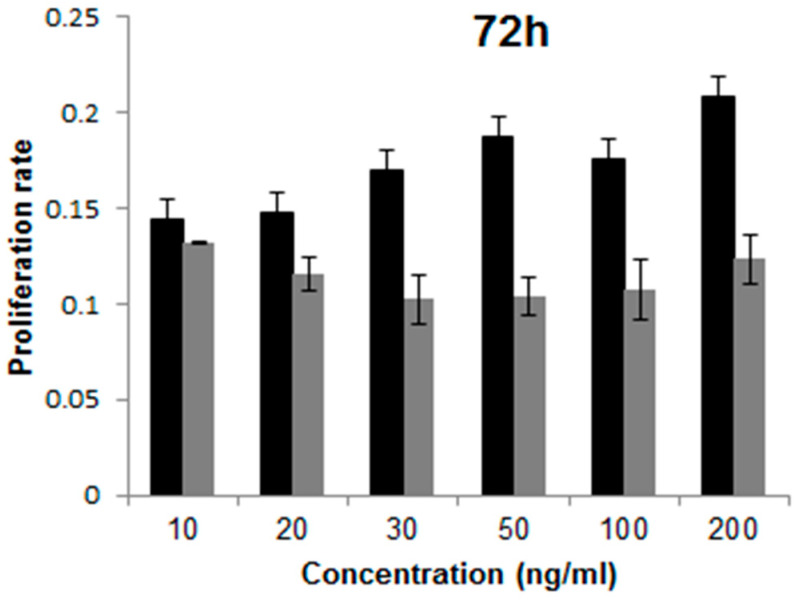
Proliferation analysis of H9 hESC cultured in homemade E8 medium containing either hFGF-2 (black bar) or hDJ-1 (gray bar) after ten passages.

**Figure 8:**
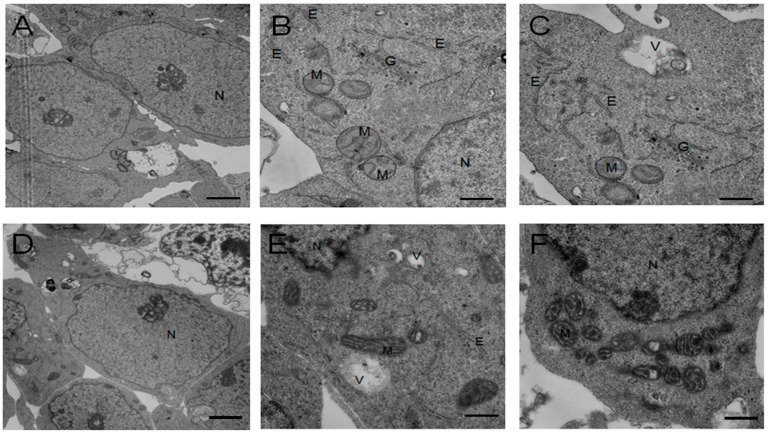
Transmission electron microscopy analysis of H9 hESCs p57 after ten passages cultured in homemade E8 medium containing hFGF-2 (**A**–**C**) or hDJ-1 (**D**–**F**). Scale bar indicates 2 µm (**A**,**D**) and 500 nm (**B**,**C**,**E**,**F**). N: nucleus, M: mitochondrion, V: vacuole, G: Golgi, E: endoplasmic reticulum.

The proliferation rate of H9 hESCs cultured with hFGF-2 or hDJ-1 was measured using CCK-8 solution (Figure 7). The cells cultured with hFGF-2 showed higher proliferation rates than those with hDJ-1 at all the tested concentrations. The cells with hFGF-1 showed the highest proliferation rate in 200 ng/mL, whereas the cells with hDJ-1 showed the highest proliferation rate in 10 ng/mL. For ultrastructural analysis of the H9 hESCs cultured with hFGF-2 or hDJ-1, the cultured cells were further analyzed using transmission electron microscopy (Figure 8). The cells cultured with hFGF-2 or hDJ-1 showed a large nucleus (Figure 8A,D). As shown in Figure 8B,C,E,F, the cells demonstrate similar Golgi, nucleus, vesicle, and endoplasmic reticulum structure. However, the cells showed some differences in mitochondrial structure. H9 hESCs cultured with hFGF-2 showed immature mitochondria structure (Figure 8B,C), which is a typical hPSCs characteristic, whereas the H9 hESCs cultured with hDJ-1 demonstrated a slightly elongated form of mitochondria with cristae structure (Figure 8E,F). However, the elongated structure of the cells’ mitochondria cultured with hDJ-1 differs from the elongated form of mitochondria with very well-developed cristae of the differentiated cells or somatic cells. 

**Figure 9:**
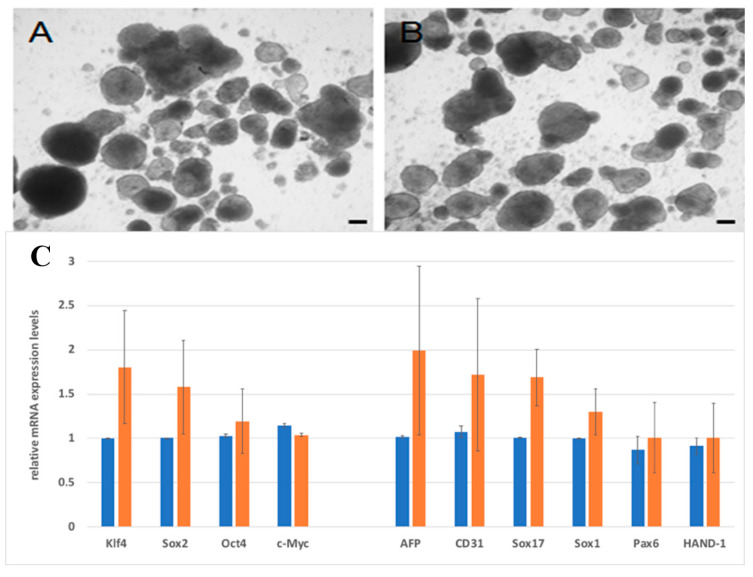
EB formation of H9 hESCs p60 after 14 days cultured in homemade E8 medium containing (**A**) hFGF-2 or (**B**) hDJ-1. Scale bar indicates 100 µm. (**C**) Gene expression profile of EBs differentiated from H9 hESCs p58 after 14 days cultured in homemade E8 medium containing hFGF-2 (blue bar) or hDJ-1 (orange bar).

The cells were subjected to differentiation assay using embryoid body (EB) formation to assess in vitro pluripotency of H9 hESCs grown with our homemade E8 media with hFGF-2 or hDJ-1 (Figure 9). After 14 days, the cells cultured in both conditions formed a round shape of the embryoid body. The EBs were further compared at a molecular level using pluripotency and three germ layer markers (Figure 9E). EBs from hESCs cultured with hDJ-1 expressed lower mRNA levels of the pluripotency markers, Oct4 and Sox2, than those with hFGF-2. For mRNA expression levels of the three germ layer markers, EBs from hDJ-1 showed higher levels of Sox1 and AFP, similar levels of Sox17, and lower levels of Pax6, CD31, and HAND-1, than EBs from hFGF-2.

**Figure 10:**
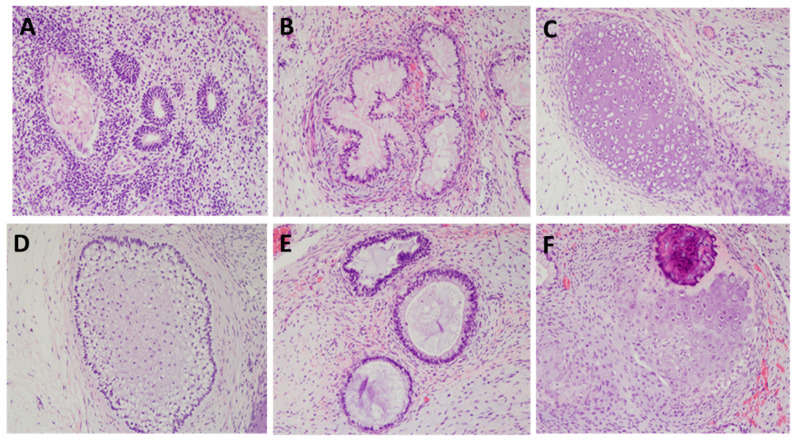
Teratoma assay. H9 hESC cultured in homemade E8 media containing either hFGF-2 or hDJ-1 were injected into NOD or NOD/SCID mice. After 12 weeks, teratomas were formed in both groups. Hematoxylin and eosin stains of teratoma microsections revealed robust differentiation into all three germ layers in the hFGF-2 group (**A**–**C**) and hDJ-1 group (**D**–**F**). (**A**) Neuroepithelium (ectoderm), (**B**) cartilage (mesoderm), (**C**) glandular tissue (endoderm), (**D**) squamous epithelium (ectoderm), (**E**) cartilage (mesoderm), and (**F**) glandular tissue (endoderm) lineages.

H9 hESCs cultured with hFGF-2 were injected into NOD mice, or hDJ-1 were injected into NOD or NOD/SCID mice to test in vivo pluripotency. After 12 weeks, teratomas were formed in three mice out of three NOD mice injected with hFGF-2 and three mice out of six NOD or NOD/SCID mice injected with hDJ-1. The teratomas were isolated and analyzed for three germ layers (Figure 10). Neuroepithelium, cartilage, and glandular epithelium structure were found in hFGF-2 teratomas. Squamous epithelium, cartilage, and glandular epithelium structure were found in hFGF-2 teratomas and hDJ-1, respectively.

## 3. Discussion

hFGF-2 promotes self-renewal and pluripotency and is an essential ingredient of many defined media including E8 for culturing hPSCs [3,18]. hPSCs expresses all FGFRs, but hFGF-2 acts mostly by binding to FGFR-1 [19]. hDJ-1 also binds to FGFR-1 although the binding site is different from that of hFGF-2 [17]. To date, there has been no report of hDJ-1 effects on hPSCs. The purpose of this study was to test whether hDJ-1 could completely replace hFGF-2 in the E8 media for the long-term culture of hPSCs in feeder-free condition. 

E8 media is the simplest defined media containing high concentration of FGF2 for maintaining hPSCs in feeder-free condition [4,5,20]. In this study, hFGF-2 was completely replaced with hDJ-1 in the homemade E8 media, and then hPSC was grown for more than 10 passages in feeder-free condition without losing its self-renewal and pluripotency. However, the possibility of hPSCs secreting its own hFGF-2 by DJ-1 treatment cannot be ruled out. In addition, the Matrigel used as the substrate might contain FGF-like growth factors [21].

It is known that hFGF-2 is structurally unstable in culture media at 37 °C and aggregated within 12 h [6,7]. Therefore, the culture media need to be changed every day, which makes hPSC culture costly and cumbersome. For higher thermal stability, hFGF-2 has been mutated [6,22,23,24], PEGylated [25], heparinized [6,26], or encapsulated in a PLGA microsphere [27]. In this study, the culture media containing hDJ-1 was changed every day to maintain the H9 cells. We tried to change the media every other day without success (data not shown). This does not necessarily mean that hDJ-1 is as unstable as hFGF-2 because there are accumulations of lactic acid, NH_4_^+^, and other toxic metabolites that make the everyday change preferable [7]. When the nominal prices of E8 media components were surveyed, hFGF-2 costs 87% of the total price [20]. The price of hDJ-1 is less than half of hFGF-2. Therefore, a significant price reduction of E8 media can be expected.

TGF-β was added in our culture media because TGF-β maintained pluripotency and led to consistent long-term culture stability of hPSCs [4,28] so that most E8 media including commercial products contain TGF-β as a necessary component [5,20]. Interestingly, E8 media without TGF-β has also been reported [29]. Instead of hFGF-2 used in the conventional hPSCs culture, human leukemia inhibitory factor (hLIF) with several small molecule inhibitors and mouse embryonic fibroblasts (MEFs) as a feeder has been used in the näive hPSC culture [30]. The primed hPSCs grown in the conventional methods are considered to be the post-implantation epiblast-derived stem cells (EpiSCs), whereas the näive hPSCs cultured using hLIF is considered to be more primitive pre-implantation epiblast. hLIF is the most pleiotropic member of the interleukin-6 family of cytokines [31]. Wnt is another factor that allows for long-term expansion of hPSCs without FGF activation [32]. The role of Wnt/β-catenin signaling in hPSC is still poorly understood and controversial because of the ambiguous role of Wnt in proliferation and differentiation. Wnt/β-catenin signaling maintains hESCs in the undifferentiated state [33,34,35]; however, it has also induced differentiation [36,37].

Not only for self-renewal and pluripotency of hPSCs, hFGF-2 can be used in other applications such as a therapeutic tool [38]. It would be interesting to see whether hDJ-1 can be used for those clinical purposes instead of hFGF-2 in the future. In addition, hFGF-2 is used for self-renewal of multipotent endodermal progenitor cells and neural progenitor cells and is also used in the differentiation of hPSCs into epiblast-like cells, primitive streak mesoderm, and mesenchymal angioblast [39]. In addition, hFGF-2 mediates developments from anterior endoderm to lung progenitors, from cardiovascular colony-forming cells to endothelial cells, from primitive streak mesoderm to chondrogenic cells, from neural progenitors to oligodendrocytes, from ectoderm to otic hair cells [39]. hFGF-2 has paradoxically opposite effects on cell proliferation and differentiation. In non-stem cells, hFGF-2 maintains energy balance and glucose homeostasis [40], acts as a neuroprotective agent under oxidative stress by controlling reactive oxygen species [41], and promotes osteogenesis and induce angiogenesis through FGFR-1 activation signaling both in vitro and in vivo [17]. Therefore, it needs to be tested in the future to see whether hDJ-1 has similar functions.

## 4. Materials and Methods

### 4.1. Materials

The H9 hESC were obtained from WiCell (Madison, WI) under a material transfer agreement. Dulbecco’s modified Eagle’s medium (DMEM), DMEM/F12 HEPES, Knockout serum replacement, E8 medium, L-glutamine, penicillin–streptomycin, non-essential amino acids, 2-mercaptoethanol, NBT/BCIP Substrate Solution, Trizol reagent, SYBR Green Real-Time PCR master mix, and colcemid KaryoMAX were purchased from Thermo Fisher Scientific (Waltham, MA, USA). L-ascorbic acid-2-phosphate, sodium selenite, sodium citrate solution, Gurr’s/Leishmann’s stain, and CCK-8 solution were from Sigma-Aldrich (St. Louis, MO, USA). Holo-transferrin and insulin were from Prospec (Rehovot, Israel). Transforming growth factor-β (TGF-β) was from GenScript (Piscataway, NJ). hFGF-2 [42] and hDJ-1 [17] were made in our laboratories. hESC-qualified Matrigel, fetal bovine serum (FBS), and ultra-low attachment plates were from Corning (Corning, NY, USA). RNase inhibitor was from Roche (Basel, Switzerland). RT-qPCR primers were from Macrogen (Seoul, Korea). The sequences of the primers are listed in Supplementary Table S1. Collagenase IV and mitomycin C were from Worthington Biochemical (Lakewood, NJ, USA). Y-27632 was from Tocris Bioscience (Bristol, UK). NaCl was from Samchun Chemical (Pyeongtaek, Korea). The cDNA Reverse Transcription Kit and Human Clariom S Assay kit for the DNA microarray were from Applied Biosystems (Waltham, MA, USA). Spurr’s embedding medium was from Electron Microscopy Science (Hatfield, PA, USA). NOD/SCID mice were from Koatech (Pyeongtaek, Korea). The antibodies were obtained from Abcam (Cambridge Science Park, UK), R&D Systems (Minneapolis, MN, USA), and Thermo Fisher Scientific (Waltham, MA, USA), as listed in Table S2. All materials were analytical grade or cell-culture grade.

### 4.2. Cell Culture and Optimization of Extracellular Factors

The H9 hESCs were cultured on mitotically arrested CF1 MEF feeder layers using 10 µg/mL of mitomycin C in knockout DMEM/F12 supplemented with 20% knockout serum replacement, 2 mM L-glutamine, 1% penicillin–streptomycin, 0.1 mM non-essential amino acid, 0.1 mM 2-mercaptoethanol, and 4 ng/mL of hFGF-2. For feeder-free culture, H9 hESCs were cultured on hESC-qualified matrigel in commercial E8 medium [5]. Once H9 hESCs attained 70–80% confluence, colonies were manually split or enzymatically split using 1 mg/mL of collagenase IV. The cells were subcultured further in E8 medium containing 10 µg/mL Y-27632. Subsequently, H9 hESCs were maintained in homemade E8 media consisting of DMEM/F12 supplemented with 64 µg/mL L-ascorbic acid-2-phosphate, 14 ng/mL sodium selenite, 10 µg/mL holo-transferrin, 20 µg/mL insulin, 1 mg/mL NaCl, 1% penicillin–streptomycin, 2 ng/mL TGF-β, and 100 ng/mL of either hFGF-2 or hDJ-1. The media was changed every day except for the next day after the split. All experiments using hESCs were conducted under the approval of the Institutional Ethics Committee, University of Ulsan College of Medicine, Seoul, Korea. 

### 4.3. Alkaline Phosphatase Staining and Immunofluorescence

Alkaline phosphatase staining was performed using 1-Step NBT/BCIP Substrate Solution. The H9 hESCs were incubated with NBT/BCIP substrate solution for 15 min at room temperature, washed three times with phosphate-buffered saline (PBS), and fixed with 4% paraformaldehyde. For the immunofluorescence study, cells were washed three times with PBS and fixed with 4% paraformaldehyde for 20 min at room temperature. After washing the cells with PBS three times, cells were permeabilized with 0.1% Triton-X for 15 min at room temperature and blocked by 1% BSA and 2% FBS in PBS containing 0.1% Triton-X for 1 h at room temperature. Subsequently, cells were incubated with primary antibodies, including rabbit polyclonal Oct4 antibody (2 µg/mL), rabbit polyclonal Sox2 antibody (2 µg/mL), and rabbit polyclonal Nanog antibody (2 µg/mL) for 1 h at room temperature. Cells were washed three times with PBS buffer and further incubated with secondary antibodies, including Alexa Fluor™ 488 conjugated goat anti-rabbit IgG H and L (1:500) and Alexa Fluor™ 594 goat anti-mouse IgG (1:500) for 1 h at room temperature, respectively. The cells were mounted in mounting medium (Vectashield), and marker expression was visualized by Zeiss fluorescent microscope.

### 4.4. Flow Cytometry Analysis

At 80–90% confluence, the H9 hESCs from all the experimental groups were harvested using 1 mg/mL of collagenase IV and passed through 0.2 µm filter to ensure single-cell dissociation. After washing the hESCs with PBS, the cells were incubated with a blocking buffer containing 1% BSA and 2% FBS in PBS for 1 h on ice. Subsequently, cells were incubated with Alexa fluor 647-conjugated mouse monoclonal SSEA4 antibody and Alexa fluor 488-conjugated mouse monoclonal TRA-1-60 antibody for 1 h on ice. Finally, incubated cells were washed three times with blocking buffer; then, cells were evaluated for corresponding marker expression using flow cytometry. 

### 4.5. Real-Time Polymerase Chain Reaction (PCR) Quantification

Total RNA was isolated using Trizol reagent and reverse transcribed into complementary DNA (cDNA) with High-Capacity cDNA Reverse Transcription Kit according to the manufacturer’s instructions. cDNA was synthesized using 2 µg of total RNA in 25 µL reaction mixture. Real-time quantitative RT-PCR assay was performed using the Applied Biosystems FAST 7500 Real-Time PCR system. All reactions were performed in triplicates using the SYBR Green Real-Time PCR master mix containing RNase inhibitor and primers. The primer sequences are shown in Table S1. All PCR data were analyzed using the delta–delta Ct method and normalized to housekeeping gene GAPDH with Applied Biosystems 7500 system SDS software. All samples were compared to their respective controls in each experiment. 

### 4.6. Karyotyping

Karyotyping analysis of H9 hESCs was conducted using standard G-banding techniques. The hESCs were treated with 0.1 mg/mL of colcemid KaryoMAX for 3 h. After centrifuging the cells, the cell pellets were resuspended in prewarmed 1% of sodium citrate solution and incubated for 50 min. The cells were pelleted by centrifugation and resuspended in fixative solution (3:1 (*v/v*) methanol/acetic acid). Metaphase spreads were prepared on glass microscope slides and G-banded by exposure to trypsin and stained with 3:1 Gurr’s/Leishmann’s stain. 

### 4.7. Proliferation Assay

The H9 hESCs were cultured on human ES-qualified matrigel in homemade E8 medium containing different concentrations of either homemade hFGF-2 or hDJ-1 ranging from 10 to 200 ng/mL (10, 20, 30, 50, 100, and 200). The ES cells were plated at a density of 50,000 cells per cm^2^ onto human ES-qualified Matrigel-coated 96-well plates, and 10 µL of the CCK-8 solution was added to each well of the plate. After incubation of plate at 37 °C for 1 h, the absorbance was measured at 450 nm using a microplate reader on day 0, 4, and 7 of hESC culture. The medium was changed daily, and Y-27632 was only used when cells were thawed and passaged. All experiments were performed in triplicates.

### 4.8. Transmission Electron Microscopy

The core serviced the transmission electron microscopy. hESCs were harvested by centrifugation, and the cell pellet was fixed by 2.5% glutaraldehyde and 2% paraformaldehyde in sodium cacodylate buffer (pH 7.2) at 4 °C. Then, the fixed specimen was postfixed in 1% osmium tetraoxide (OsO4) containing 1.5% potassium ferrocyanide for 30 min at 4 °C. The fixed specimen was dehydrated using an ethanol series of 50%, 60%, 70%, 80%, 90%, 100%, and 100% for 20 min in each. The specimen was subsequently transferred to Spurr’s embedding medium. After impregnating the specimen with the pure resin, the tissue specimens were embedded in the same resin mixture, and samples were sectioned (60–70 nm) using a Leica UltracutUCT ultramicrotome (GmbH, Austria) and double-stained first with 2% uranyl acetate for 20 min and then with lead citrate for 10 min. Then, the sections were observed under the Hitachi H7600 transmission electron microscope at 80 kV.

### 4.9. DNA Microarray

A DNA microarray experiment was carried out by Macrogen (Seoul, Korea). Total cDNA was synthesized using the GeneChip Whole Transcript Amplification kit as described by the manufacturer. Then, the sense cDNA was fragmented and biotin-labeled with terminal deoxynucleotidyl transferase using the GeneChip WT Terminal labeling kit. Approximately 5.5 μg of labeled DNA target was hybridized to the Affymetrix GeneChip Array at 45 °C for 16 hrs. Hybridized arrays were washed and stained on a GeneChip Fluidics Station 450 and scanned on an Affymetrix GCS3000 Scanner. Array data export processing and analysis were performed using Affymetrix GeneChip Command Console Software. Affymetrix Power Tools and R 3.1.2 were used for the analysis. 

Hierarchical clustering was done by the complete linkage Euclidean method. Gene enrichment and functional annotation analysis were performed using Gene Ontology (GO) and Kyoto Encyclopedia of Genes and Genomes (KEGG) pathway. Significance was calculated by modified fisher’s exact test.

### 4.10. Differentiation Assay

The H9 hESC colonies were dissociated into small pieces for spontaneous differentiation assay and seeded as spheres on ultra-low attachment plates in homemade E8 medium for seven days to form embryoid bodies. The embryoid bodies were further differentiated for seven days in Knockout DMEM supplemented with 2 mM L-glutamine, 20% FBS, 1% penicillin–streptomycin, 0.1 mM non-essential amino acid, 0.1 mM 2-mercaptoethanol. Total RNA was isolated from embryoid bodies, and real-time PCR analysis was performed as described above.

### 4.11. Teratoma Assay

All animal experiments were reviewed and approved by the Institutional Animal Care and Use Committee (IACUC) of Asan Institute for Life Sciences, Asan Medical Center. The committee abides by the Institute of Laboratory Animal Resources (ILAR) guide. For teratoma formation experiments, the H9 hESCs were digested with 1 mg/mL of collagenase IV and filtered through 0.2 µm filter to ensure single-cell dissociation. The cell pellets were resuspended in DMEM/F12 medium, and 1 × 10⁶ cells were subcutaneously injected into a young (7-week-old) NOD/SCID mouse. Three animals were used for each medium. Teratoma growth was measured every week, and after eight weeks of injection, teratomas were dissected, measured, and fixed with PBS containing 4% paraformaldehyde. The paraffin-embedded tumors were sliced, and the sections were stained with hematoxylin and eosin. All animal experiments were performed at the Asan medical center’s infection-free animal facility following the ethical committee’s approval and the animal protocol.

### 4.12. Statistical Analysis

The statistical significance of the responses was performed by a two-tailed Student’s *t*-test. Values of *p* < 0.05 were considered to indicate statistical significance. All data are presented as the mean ± standard error (SE) of *n* ≥ 3 of 3 independent experiments. 

## 5. Conclusions

In conclusion, hDJ-1 can maintain the pluripotency of hPSCs without hFGF2 in the culture media in feeder-free conditions, which was deduced from the stem cell markers’ results, karyotyping, DNA microarray, transmission electron microscopy, in vitro differentiation assay, and the teratoma assay.

## Data Availability

The datasets generated and analyzed during the present study are available from the corresponding author on reasonable request.

## Supplementary Materials

The following are available online at https://www.mdpi.com/article/10.3390/ijms22115954/s1.

## Significance Statement

DJ-1 is known to bind to FGFR at a site different from FGF-2. Human pluripotent stem cell can be grown in a defined medium with DJ-1 without FGF-2 in feeder-free conditions, which was deduced from the stem cell markers’ results, karyotyping, DNA microarray, transmission electron microscopy, in vitro differentiation assay, and the teratoma assay. Therefore, DJ-1 can replace or supplement expensive FGF-1 in maintenance of human pluripotent stem cell.
