# Supplementary material for: DJ-1 Can Replace FGF-2 for Long-Term Culture of Human Pluripotent Stem Cells in Defined Media and Feeder-Free Condition
Source: Int J Mol Sci. 2021 May 31;22(11):5954. doi: 10.3390/ijms22115954 (PMC8197809; doi:10.3390/ijms22115954)
Supplement: Supplementary file 1 [file ijms-22-05954-s001.zip › Supplementary Information.pdf]

Supplementary Information

Supplementary Table 1. RT-qPCR primer sequences

Gene	Forward primer	Reverse primer
GAPDH	5'-CTGGTAAAGTGGATATTGTTGCCAT-3'	5'-TGGAATCATATTGGAACATGTAAACC-3'
Oct4	5'-GTGGAGGAAGCTGACAACAA-3'	5'-ATTCTCCAGGTTGCCTCTCA-3'
Nanog	5'-CCTGTGATTTGTGGGCCTG-3'	5'-GACAGTCTCCGTGTGAGGCAT-3'
Sox2	5'-GGGAAATGGGAGGGGTGCAAAAGAGG-3'	5'-TTGCGTGAGTGTGGATGGGATTGGTG-3'
c-Myc	5'-GCGTCCTGGGAAGGGAGATCCGGAGC-3'	5'-TTGAGGGGCATCGTCGCGGGAGGCTG-3'
Klf4	5'-TGATTGTAGTGCTTTCTGGCTGGGCTCC-3'	5'-ACGATCGTGGCCCCGGAAAAGGACC-3'
Sox17	5'-CGCTTTCATGGTGTGGGCTAAGGACG-3'	5'-TAGTTGGGGTGGTCCTGCATGTGCTG-3'
AFP	5'-GAATGCTGCAAACTGACCACGCTGGAAC-3'	5'-TGGCATTCAAGAGGGTTTTCAGTCTGGA-3'
PAX6	5'-ACCCATTATCCAGATGTGTTTGCCCGAG-3'	5'-ATGGTGAAGCTGGGCATAGGCGGCAG-3'
DJ-1	5'-GCTTCCAAAAGAGCTCTGGTC-3'	5'-ACATCACGGCTACACTG-3'
Pecam1	5'-TCAGGCAACGCACAAAACAG-3'	5'-GACCTGCTCGGTTCTCTCTG-3'
HAND-1	5'-TATAAGCCAGATCCGCAGGG-3'	5'-CGATGTAGCTGGTGGCTAGG-3'
Sox1	5'-AATACTGGAGACGAACGCCG-3'	5'-AGTGCTTGGACCTGCCTTAC-3'

Supplementary Table 2. The antibody information

Name	Company	Catalogue number	Dilution
Oct4	abcam	ab19857	ICC/IF 1/100<br>WB 1:1000
Sox2	abcam	ab97959	ICC/IF 1/100<br>WB 1:1000
Nanog	abcam	ab21624	ICC/IF 1/100<br>WB 1:1000
DJ-1	R&D System	925805R	ICC/IF 1:20<br>WB 1:500
SSEA4	Invitrogen	SSEA421	FACS 1:50, 1:100
Tra1-60	Invitrogen	MA1-023-D488X	FACS 1:50, 1:100

Supplementary Table 3. Preprocessed microarray data.

Image analysis, extract raw data, and analysis were done by Affymetrix Power Tools (background correction, summarization, normalization). The data were filtered by main or consensus probes.

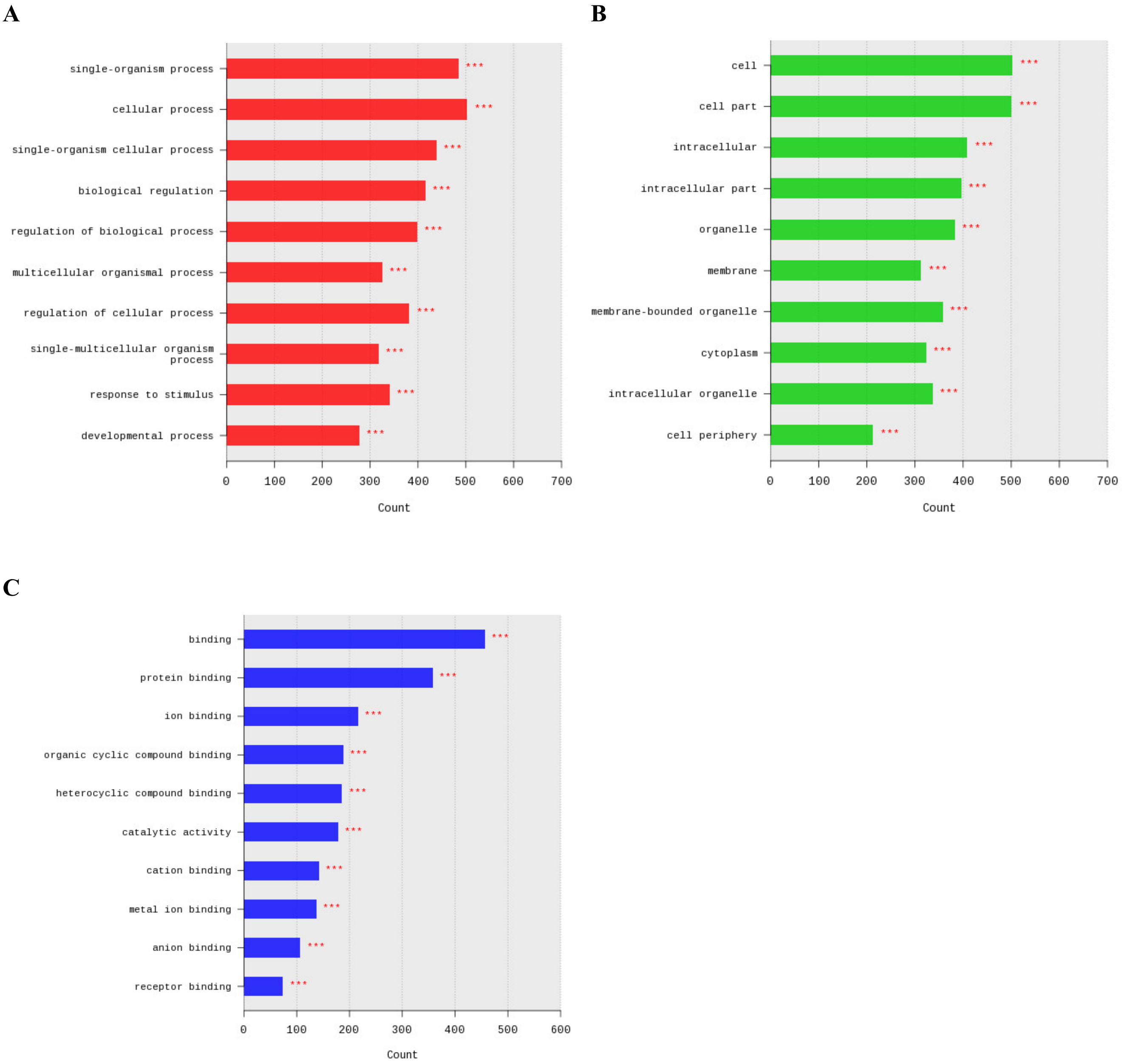

Supplementary Figure 1. Top 10 terms of GO functional analysis

A. Biological process. B. Cellular component. C. Molecular Function. P value < 0.001 (***)

Supplementary Figure 2A

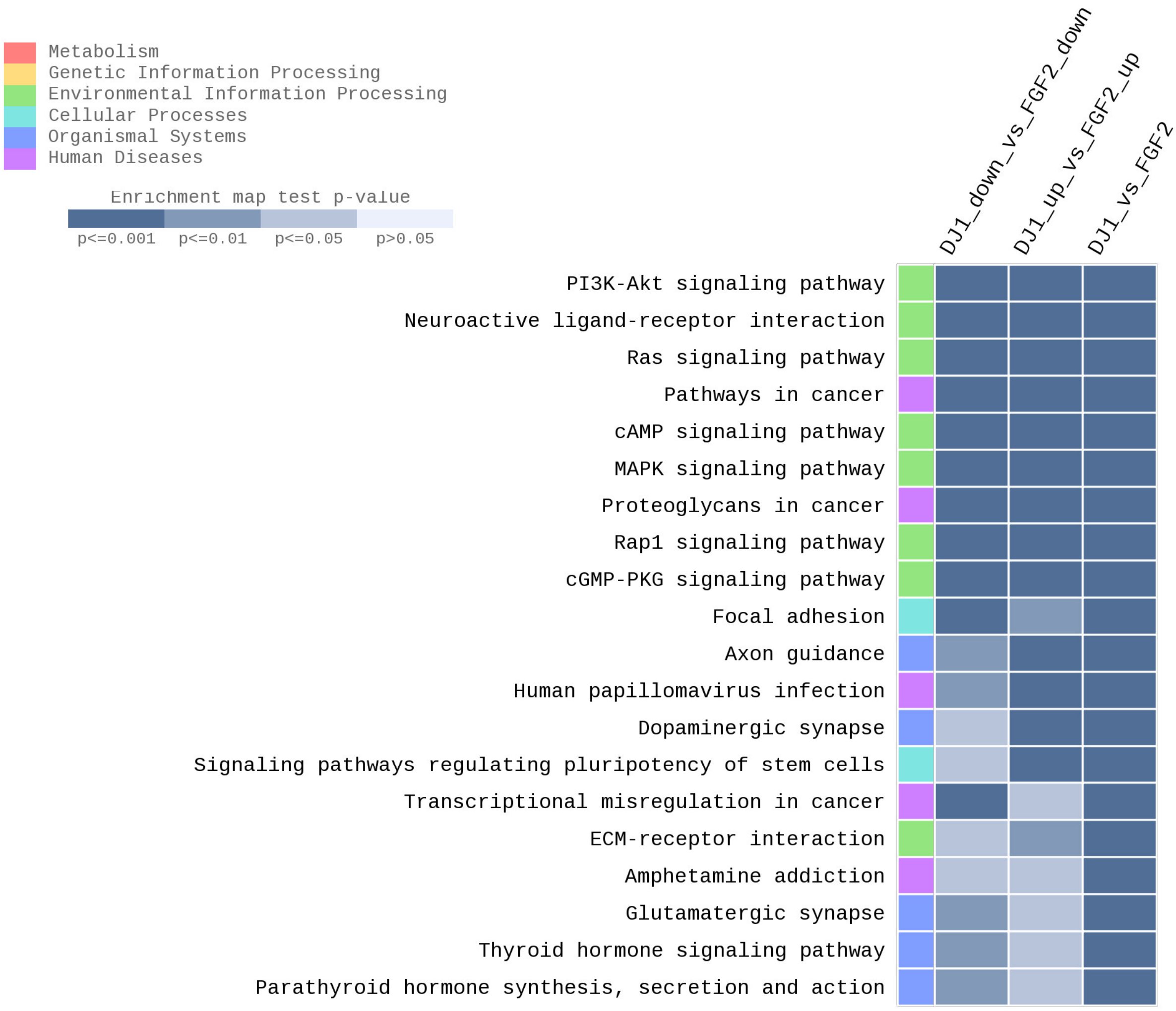

Supplementary Figure 2. KEGG gene-enrichiment heatmaps

A. Sorted. B. Filtered. C. Total. DJ1_down_vs_FGF2_down, The expressions in DJ1 media were down more than 2-fold; DJ1_up_vs_FGF2_up, The expressions in DJ1 media were up more than 2-fold; DJ1_vs_FGF2, The expressions were different more than 2-fold.
