# Supplementary figures and images for: DJ-1 Can Replace FGF-2 for Long-Term Culture of Human Pluripotent Stem Cells in Defined Media and Feeder-Free Condition
Source: Int J Mol Sci. 2021 May 31;22(11):5954. doi: 10.3390/ijms22115954 (PMC8197809; doi:10.3390/ijms22115954)

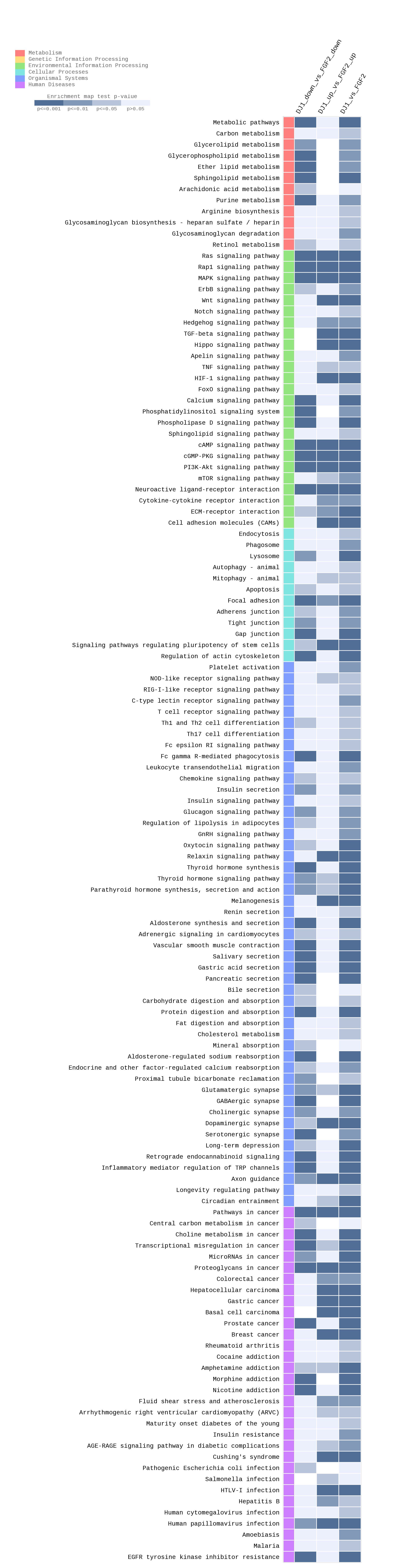

Supplement: Supplementary file 1 [file ijms-22-05954-s001.zip › Supplementary Figure 2B.png]
